# A Case of Malignant Brenner Tumor of the Ovary Incidentally Found in a Patient with Deep Vein Thrombosis

**DOI:** 10.7759/cureus.37176

**Published:** 2023-04-05

**Authors:** Wendolin J Ortiz, Michael W Morris, Mario Cervantes

**Affiliations:** 1 General Surgery, Universidad Autónoma de Baja California, Mexicali, MEX; 2 General Surgery, HCA (Hospital Corporation of America) Houston Healthcare Kingwood, Houston, USA; 3 General Surgery, United Memorial Medical Center, Houston, USA; 4 Pathology, HCA (Hospital Corporation of America) Houston Healthcare West, Houston, USA; 5 Pathology, HCA (Hospital Corporation of America) Houston Healthcare Pearland, Houston, USA; 6 Pathology, United Memorial Medical Center, Houston, USA

**Keywords:** transitional cell carcinoma, ct scan, deep vein thrombosis, brenner tumor, malignant brenner tumor

## Abstract

We present the case of a 73-year-old woman who was incidentally found to have a malignant Brenner tumor (MBT) of the ovary during an evaluation for deep vein thrombosis (DVT). The patient presented with swelling in her left leg, non-healing ulcers, weakness, and numbness in her lower limbs. Imaging studies revealed a large multiloculated cystic mass with areas of calcification in the left adnexa extending to the upper abdomen toward the gallbladder fossa. The patient underwent exploratory laparotomy with removal of the ovarian cyst, later diagnosed as a focal MBT in a background of borderline Brenner tumor. Brenner tumors of the ovary are a rare subtype of ovarian neoplasm that accounts for less than 2% of all ovarian tumors. MBTs are even rarer, comprising less than 5% of all Brenner tumors. To our knowledge, this is the first reported case of an MBT incidentally found in a patient with DVT.

## Introduction

Malignant Brenner tumors (MBT) are an uncommon and aggressive type of ovarian neoplasms that make up less than 5% of all Brenner tumors and less than 1% of all ovarian malignancies. Brenner tumors are solid, usually unilateral ovarian tumors made up of abundant stroma, which contain nests of transitional-type epithelium similar to that of the urinary tract. These nests may occasionally be cystic and lined with columnar mucus-secreting cells. Brenner tumors are categorized into benign, borderline or proliferative, and malignant subtypes, with MBT being particularly rare and characterized by stromal invasion under the microscope.

MBT usually affects women during the perimenopausal and postmenopausal periods, and symptoms such as abdominal distention, pelvic discomfort, or postmenopausal bleeding may be present. Surgical excision and pathological evaluation are necessary to diagnose MBT because it lacks specific imaging features. Treatment consists of surgical resection, and the prognosis of MBT is generally favorable [1-4].

## Case presentation

A 73-year-old African American woman with a medical history of hypertension, hyperlipidemia, type 2 diabetes, and four cerebrovascular accidents presented to the hospital with a recent diagnosis of left lower extremity deep vein thrombosis (DVT). The patient reported experiencing swelling in her left leg for the past few months, along with non-healing ulcers in both legs and weakness and numbness in her lower limbs.

On physical examination, the patient had 2+ pitting edema and small ulcers in her lower extremities. Dorsalis pedis pulses and posterior tibial pulses were bilaterally absent. An abdominal examination revealed present bowel sounds and a soft, non-tender, and distended abdomen. Venous and arterial Doppler of bilateral lower extremities confirmed the diagnosis of DVT. The patient was already on anticoagulation, and a venogram and thrombectomy were planned due to the edema and pain.

**Figure 1:**
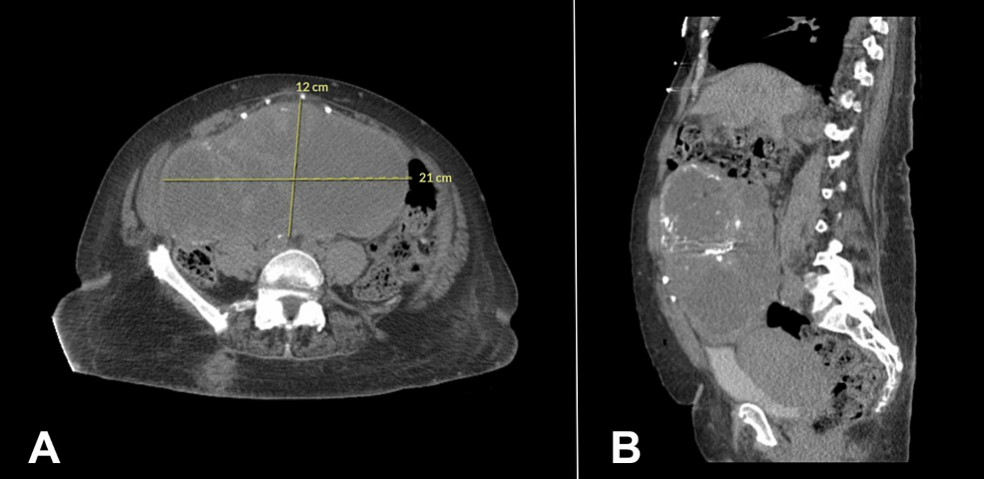
CT of the abdomen and pelvis without contrast Large multiloculated cystic mass with areas of calcification within and multiple septations extending from the left adnexa to the upper abdomen shown on the transverse (A) and sagittal (B) planes CT: computed tomography

A week later, the patient was brought to the hospital due to sudden onset tachycardia. She had a pulse rate of 140 s and was admitted for cardiovascular event evaluation. The patient's organs and systems were reviewed, and she was negative for chills or fever, vision disturbances, cough, and shortness of breath. However, she did have bilateral leg edema, left leg pain, urinary frequency, and incontinence. A computed tomography (CT) scan of the chest was obtained to ensure there was no pulmonary embolism. Although there was no evidence of acute pulmonary embolism, an 8.8 cm cystic area lesion was visualized in the anterior upper abdomen. A CT scan of the abdomen and pelvis revealed left mild hydronephrosis and a large multiloculated cystic mass with areas of calcification within and multiple septations extending from the left adnexa to the upper abdomen toward the gallbladder fossa, measuring 12 cm anterior-posterior, 21 cm transverse, and at least 20 cm in height (Figure 1).

Four days later, the patient underwent bilateral femoral, common femoral, external, and common iliac vein thrombectomy angioplasty. During the angioplasty procedure of the inferior vena cava, a 12 x 40 mm balloon was used, and it was observed that the pre-treatment stenosis was 50%, which improved to 40% post-treatment. However, there was some indication of external compression caused by the pelvic mass. During the next two days, the patient presented with pain in the right upper quadrant on deep palpation and a decreased appetite.

General surgery was consulted; however, the exploratory laparotomy was postponed due to the patient's medication regimen for deep vascular problems, including enoxaparin, clopidogrel, and aspirin. The patient returned the following month for her scheduled exploratory laparotomy with the removal of the large ovarian cyst. On examination, the abdomen was soft, with positive bowel sounds and no palpable megaly, but a large mass was observed in the mid epigastric area that was slightly tender on movements and radiated to the left upper and lower quadrants.

**Figure 2:**
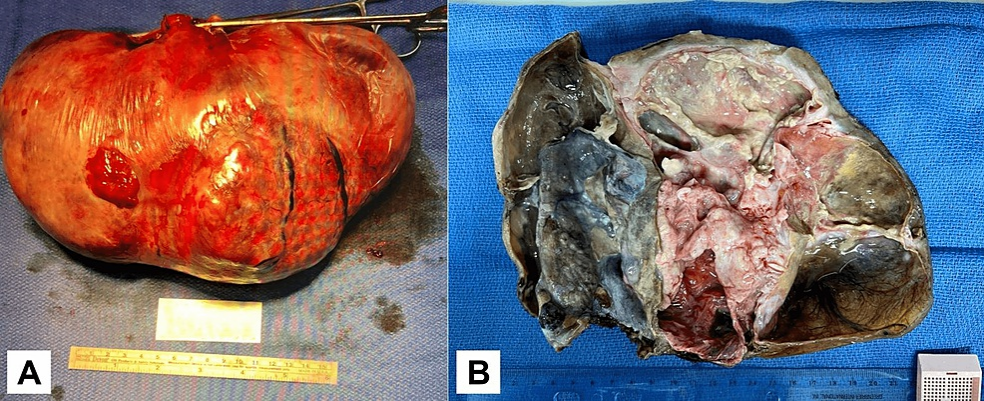
Gross appearance of the Brenner tumor A) The Brenner tumor measured approximately 10 x 8 inches in size, B) Upon gross examination, the tumor was found to be a large cystic mass, measuring 21 x 13 x 9.5 cm, with loculated yellowish secretion/debris visible on the cut section.

The patient was taken to the operating room. During the surgery, an upper midline incision was made, the abdomen was emptied and a very large mass was evident. It became obvious that it was attached to the left ovary and that it was probably a left ovarian mass. It was then excised, dividing the ovarian tissue with the mass very close to the uterus. The mass was then totally removed, inspected, and opened on the back table. It was found to be very large, about 10 x 8 inches in size (Figure 2A). The patient tolerated the procedure well and was sent to the recovery room in good, stable condition.

The gross examination revealed a large cystic tumor measuring 21 x 13 x 9.5 cm. On a cut section, it was loculated with yellowish secretion/debris. The capsule/wall thickness ranged from 0.5 to 3.5 cm, and the wall was partly fibrotic with scattered calcification (Figure 2B).

**Figure 3:**
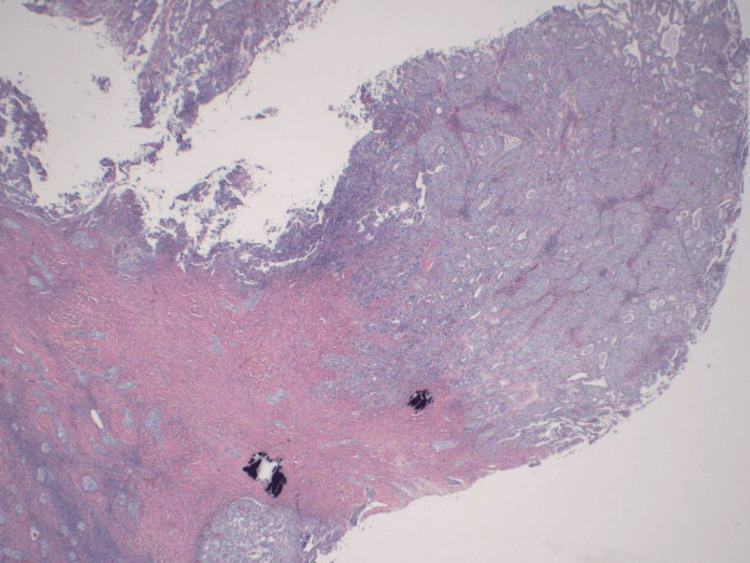
Histological image of the Brenner tumor and transitional cell carcinoma with invasion Brenner tumor (left) and transitional cell carcinoma with invasion (right) (HE, 20x) HE: Hematoxylin-eosin

**Figure 4:**
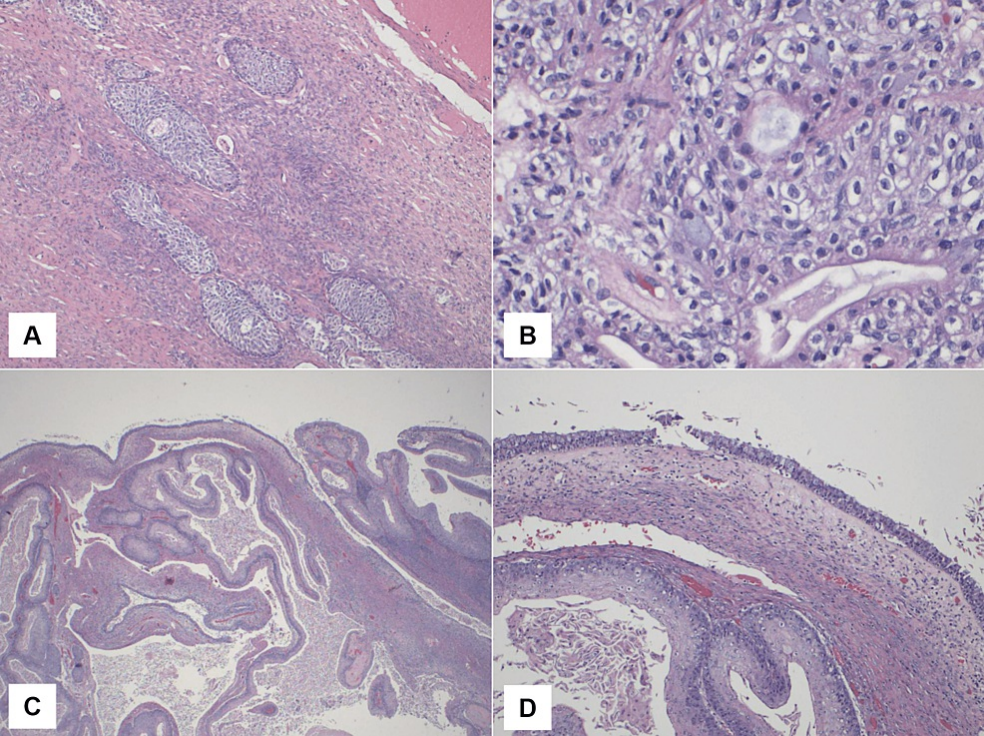
Histological features of the Brenner tumor with transitional cell islands and squamous differentiation A) Typical Brenner tumor with transitional cell islands and fibrous stroma (H&E, 100x); B) Nuclear grooves characteristic of Brenner tumors (H&E, 400x); C) Cystic area within the Brenner tumor with a mixture of squamous, columnar, and goblet cells (H&E, 20x); D) Squamous differentiation (below), a mixture of columnar with goblet cells (above) (H&E, 100x) H&E: Hematoxylin-eosin

**Figure 5:**
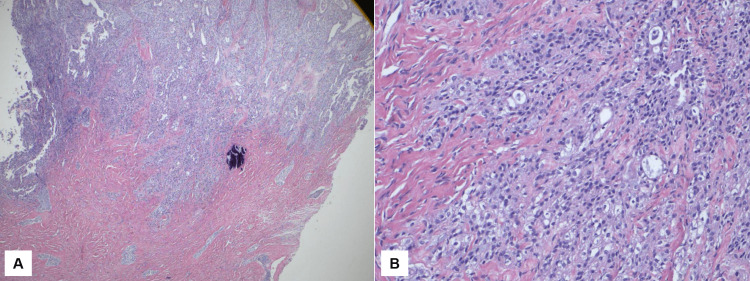
Histological evidence of tumor invasion into surrounding tissue Hematoxylin-eosin (HE) stain of invasion of the tumor cells into the surrounding tissue at 40x magnification (A) and at 200x (B)

On histology, focal microinvasion adjacent to a predominantly non-invasive papillary transitional carcinoma-like area was found, along with squamous epidermal inclusion cysts, glandular cysts with partial mucinous differentiation, and classic Brenner's tumor with fibrosis. Some of the cysts were partly ruptured with sub-epithelial chronic inflammation, histiocytosis, and fibrosis. There was also scattered dystrophic calcification (Figures 3-5). The final diagnosis was a focal MBT in a background of borderline Brenner tumor.

The patient was discharged in stable condition.

## Discussion

MBT is an uncommon type of ovarian malignancy and is usually detected incidentally during imaging for other conditions. The tumor is characterized by the presence of malignant and benign/borderline Brenner tumor components, with clear stromal invasion by the malignant epithelial components. The absence of hemorrhage or necrosis, distinct nuclear grooves with urothelial marker expression, and elevated CA125 in some patients are among the diagnostic features of MBT. However, distinguishing MBT from transitional cell carcinoma (TCC), another aggressive high-grade neoplasm with similar histologic features, can be challenging. The histological features of TCC include a high mitotic rate, severe nuclear atypia, and extensive necrosis. Immunohistochemical markers, such as CK7, p16, and p63, can aid in the differentiation of MBT and TCC [2,3].

There are other types of ovarian tumors, such as serous, mucinous, endometrioid, and clear cell carcinomas, as well as benign Brenner tumors, teratomas, and metastases from urinary tract tumors, which should be considered in the differential diagnosis of MBT. Metastasis from other organs, such as the urinary bladder or colorectum, should also be ruled out [4].

Patients with MBT typically experience symptoms similar to those of other epithelial ovarian carcinomas, such as abdominal pain, distension, and urinary urgency. Imaging studies can assist in the preoperative differential diagnosis of malignant and benign Brenner tumors, and MBTs usually contain both solid and cystic components. Most MBTs are unilateral, high-grade tumors with a median size of 10 cm, and most patients present with localized disease [5,6].

Surgery is the standard of care for all epithelial ovarian tumors, including MBT. Although the prognosis for MBT is generally good, there is currently no standardized chemotherapy regimen for the treatment of MBT due to the limited number of reported cases [1,2].

## Conclusions

Thorough diagnostic evaluation is crucial in patients who present with symptoms related to DVT, as emphasized in this case report. The incidental finding of a large ovarian cystic mass during the review of the patient's cardiovascular symptoms ultimately led to the diagnosis of MBT, a rare subtype of ovarian cancer. The successful removal of the tumor through surgery underscores the importance of early detection and prompt treatment.
